# Early mobilisation in critically ill COVID-19 patients: a subanalysis of the ESICM-initiated UNITE-COVID observational study

**DOI:** 10.1186/s13613-023-01201-1

**Published:** 2023-11-14

**Authors:** Philipp Kloss, Maximilian Lindholz, Annette Milnik, Elie Azoulay, Maurizio Cecconi, Giuseppe Citerio, Thomas De Corte, Frantisek Duska, Laura Galarza, Massimiliano Greco, Armand R. J. Girbes, Jozef Kesecioglu, Johannes Mellinghoff, Marlies Ostermann, Mariangela Pellegrini, Jean-Louis Teboul, Jan De Waele, Adrian Wong, Stefan J. Schaller, for the ESICM UNITE COVID Investigators, Buenos Aires, Alicia Gira, Philipp Eller, Tarikul Hamid, Injamam Ull Haque, Wim De Buyser, Antonella Cudia, Daniel De Backer, Pierre Foulon, Vincent Collin, Jolien Van Hecke, Elisabeth De Waele, Claire Van Malderen, Jean-Baptiste Mesland, Patrick Biston, Michael Piagnerelli, Lionel Haentjens, Nicolas De Schryver, Jan Van Leemput, Philippe Vanhove, Pierre Bulpa, Viktoria Ilieva, David Katz, Alexandra Binnie, Anna Geagea, Fernando Tirapegui, Gustavo Lago, Jerónimo Graf, Rodrigo Perez-Araos, Patricio Vargas, Felipe Martinez, Eduardo Labarca, Daniel Molano Franco, Daniela Parra-Tanoux, David Yepes, Ahmed Hammouda, Omar Elmandouh, Ahmed Azzam, Aliae Mohamed Hussein, Islam Galal, Ahmed K. Awad, Mohammed A. Azab, Maged Abdalla, Hebatallah Assal, Mostafa Alfishawy, Sherief Ghozy, Samar Tharwat, Abdullah Eldaly, Anneli Ellervee, Veronika Reinhard, Anne Chrisment, Chrystelle Poyat, Julio Badie, Fernando Berdaguer Ferrari, Björn Weiss, Clara Schellenberg, Julius J Grunow, Marco Lorenz, Stefan J Schaller, Peter Spieth, Marc Bota, Falk Fichtner, Kristina Fuest, Tobias Lahmer, Johannes Herrmann, Patrick Meybohm, Nikolaos Markou, Georgia Vasileiadou, Evangelia Chrysanthopoulou, Panagiotis Papamichalis, Ioanna Soultati, Sameer Jog, Kushal Kalvit, Sheila Nainan Myatra, Ivan Krupa, Aisa Tharwat, Alistair Nichol, Aine McCarthy, Ata Mahmoodpoor, Tommaso Tonetti, Paolo Isoni, Savino Spadaro, Carlo Alberto Volta, Lucia Mirabella, Alberto Noto, Gaetano Florio, Amedeo Guzzardella, Chiara Paleari, Federica Baccanelli, Marzia Savi, Massimo Antonelli, Gennaro De Pascale, Barbara Vaccarini, Giorgia Montrucchio, Gabriele Sales, Katia Donadello, Leonardo Gottin, Marta Nizzero, Enrico Polati, Silvia De Rosa, Demet Sulemanji, Abdurraouf Abusalama, Muhammed Elhadi, Montelongo Felipe De Jesus, Daniel Rodriguez Gonzalez, Victor Hugo Madrigal Robles, Nancy Canedo, Alejandro Esquivel Chavez, Tarek Dendane, Bart Grady, Ben de Jong, Eveline van der Heiden, Patrick Thoral, Bas van den Bogaard, Peter E. Spronk, Sefanja Achterberg, Melanie Groeneveld, Ralph K. L. So, Calvin de Wijs, Harm Scholten, Albertus Beishuizen, Alexander D. Cornet, Auke C. Reidinga, Hetty Kranen, Roos Mensink, Sylvia den Boer, Marcel de Groot, Oliver Beck, Carina Bethlehem, Bas van Bussel, Tim Frenzel, Celestine de Jong, Rob Wilting, Jannet Mehagnoul-Schipper, Datonye Alasia, Ashok Kumar, Ahad Qayyum, Muhammad Rana, Mustafa Abu Jayyab, Rosario Quispe Sierra, Aaron Mark Hernandez, Lúcia Taborda, Mónica Anselmo, Tiago Ramires, Catarina Silva, Carolina Roriz, Rui Morais, Pedro Póvoa, Patricia Patricio, André Pinto, Maria Lurdes Santos, Vasco Costa, Pedro Cunha, Celina Gonçalves, Sandra Nunes, João Camões, Diana Adrião, Ana Oliveira, Ali Omrani, Muna Al Maslamani, Abdurrahmaan Suei elbuzidi, Bara Mahmoud Al qudah, Abdel Rauof Akkari, Mohamed Alkhatteb, Anas Baiou, Ahmed Husain, Mohamed Alwraidat, Ibrahim Abdulsalam Saif, Dana Bakdach, Amna Ahmed, Mohamed Aleef, Awadh Bintaher, Cristina Petrisor, Evgeniy Popov, Ksenia Popova, Mariia Dementienko, Boris Teplykh, Alexey Pyregov, Liubov Davydova, Belskii Vladislav, Elena Neporada, Ivan Zverev, Svetlana Meshchaninova, Dmitry Sokolov, Elena Gavrilova, Irina Shlyk, Igor Poliakov, Marina Vlasova, Ohoud Aljuhani, Amina Alkhalaf, Felwa Bin Humaid, Yaseen Arabi, Ahmed Kuhail, Omar Elrabi, Madihah E. Ghannam, Amit Kansal, Vui Kian Ho, Jensen Ng, Raquel Rodrígez García, Xiana Taboada Fraga, Mª del Pilar García-Bonillo, Antonio Padilla-Serrano, Marta Martin Cuadrado, Carlos Ferrando, Ignacio Catalan-Monzon, Fernando Frutos-Vivar, Jorge Jimenez, Carmen Rodríguez-Solis, Enric Franquesa-Gonzalez, Guillermo Pérez Acosta, Luciano Santana Cabrera, Juan Pablo Aviles Parra, Francisco Muñoyerro Gonzalez, Maria Lorente del Carmen Conesa, Ignacio Yago Martinez Varela, Orville Victoriano Baez Pravia, Maria Cruz Martin Delgado, Carlos Munoz de Cabo, Ana-Maria Ioan, Cesar Perez-Calvo, Arnoldo Santos, Ane Abad-Motos, Javier Ripolles-Melchor, Belén Civantos Martin, Santiago Yus Teruel, Juan Higuera Lucas, Aaron Blandino Ortiz, Raúl de Pablo Sánchez, Jesús Emilio Barrueco-Francioni, Lorena Forcelledo Espina, José M. Bonell-Goytisolo, Iñigo Salaverria, Antonia Socias Mir, Emilio Rodriguez-Ruiz, Virginia Hidalgo Valverde, Patricia Jimeno Cubero, Francisca Arbol Linde, Nieves Cruza Leganes, Juan Maria Romeu, Pablo Concha, José Angel Berezo-Garcia, Virginia Fraile, Cristina Cuenca-Rubio, David Pérez-Torres, Ainhoa Serrano, Clara Martínez Valero, Andrea Ortiz Suner, Leire Larrañaga, Noemi Legaristi, Gerardo Ferrigno, Safa Khlafalla, Rosita Bihariesingh-Sanchit, Frank Zoerner, Jonathan Grip, Kristina Kilsand, Johan Mårtensson, Jonas Österlind, Magnus von Seth, Johan Berkius, Samuele Ceruti, Andrea Glotta, Seval Izdes, Işıl Özkoçak Turan, Ahmet Cosar, Burcin Halacli, Necla Dereli, Mehmet Yilmaz, Türkay Akbas, Gülseren Elay, Selin Eyüpoğlu, Yelíz Bílír, Kemal Tolga Saraçoğlu, Ebru Kaya, Ayca Sultan Sahin, Pervin Korkmaz Ekren, Tuğçe Mengi, Kezban Ozmen Suner, Yakup Tomak, Ahmet Eroglu, Asad Alsabbah, Katie Hanlon, Kevin Gervin, Sean McMahon, Samantha Hagan, Caroline V Higenbottam, Randeep Mullhi, Lottie Poulton, Tomasz Torlinski, Allen Gareth, Nick Truman, Gopal Vijayakumar, Chris Hall, Alasdair Jubb, Lenka Cagova, Nicola Jones, Sam Graham, Nicole Robin, Amanda Cowton, Adrian Donnelly, Natalia Singatullina, Melanie Kent, Carole Boulanger, Zoë Campbell, Elizabeth Potter, Natalie Duric, Tamas Szakmany, Orinta Kviatkovske, Nandor Marczin, Caroline Ellis, Rajnish Saha, Chunda Sri-Chandana, John Allan, Lana Mumelj, Harish Venkatesh, Vera Nina Gotz, Anthony Cochrane, Barbara Ficial, Shruthi Kamble, Nuttha Lumlertgul, Christopher Oddy, Susan Jain, Giulia Beatrice Crapelli, Aikaterini Vlachou, David Golden, Sweyn Garrioch, Jeremy Henning, Gupta Loveleena, Miriam Davey, Lina Grauslyte, Erika Salciute-Simene, Martin Cook, Danny Barling, Phil Broadhurst, Sarah Purvis, Michael Spivey, Benjamin Shuker, Irina Grecu, Daniel Harding, Natalia Singatullina, James T. Dean, Nathan D. Nielsen, Sama Al-Bayati, Mohammed Al-Sadawi, Mariane Charron, Peter Stubenrauch, Jairo Santanilla, Catherine Wentowski, Dorothea Rosenberger, Polikseni Eksarko, Randeep Jawa

**Affiliations:** 1 Charité - Universitätsmedizin Berlin, corporate Member of Freie Universität Berlin and Humboldt-Universität zu Berlin, Department of Anesthesiology and Intensive Care Medicine (CCM/CVK), Berlin, Germany; 2 Research Platform Molecular and Cognitive Neurosciences (MCN), Department of Biomedicine, University of Basel, Basel, Switzerland; 3 Médecine Intensive et Réanimation, APHP, Saint-Louis Hospital, Paris University, Paris, France; 4 Université de Paris, Paris, France; 5 Department of Biomedical Sciences, Humanitas University, Via Rita Levi Montalcini 4, Pieve Emanuele, 20072 Milan, Italy; 6 IRCCS Humanitas Research Hospital, Via Manzoni 56, Rozzano, 20089 Milan, Italy; 7 School of Medicine and Surgery, University of Milano-Bicocca, Milan, Italy; 8 Department Neuroscience, Neurointensive Care, IRCCS Fondazione San Gerardo dei Tintori, Monza, Italy; 9 Department of Internal Medicine and Pediatrics, Faculty of Medicine and Health Sciences, Ghent University, Ghent, Belgium; 10 Department of Intensive Care Medicine, Ghent University Hospital, Ghent, Belgium; 11 Department of Anaesthesia and Intensive Care, Third Faculty of Medicine, Charles University, Prague, Czech Republic; 12 FNKV University Hospital in Prague, Prague, Czech Republic; 13 Intensive Care Unit, Hospital General Universitario de Castellón, Castellón de La Plana, Spain; 14 Department of Intensive Care Medicine, Research VUmc Intensive Care (REVIVE), Amsterdam Medical Data Science (AMDS), Amsterdam Cardiovascular Sciences (ACS), Amsterdam Infection and Immunity Institute (AI&II), UMC, Location VUmc, VU Amsterdam, Amsterdam, The Netherlands; 15 Department of Intensive Care Medicine, University Medical Center Utrecht, Utrecht University, Utrecht, The Netherlands; 16 School of Sports and Health Sciences, University of Brighton, Brighton, UK; 17 Department of Critical Care, King’s College London, Guy’s & St Thomas’ Hospital, London, UK; 18 Intensive Care Unit, AnOpIVA, Akademiska Sjukhuset, Uppsala, Sweden; 19 Hedenstierna Laboratory, Department of Surgical Science, Uppsala University, Uppsala, Sweden; 20 Service de Médecine Intensive-Réanimation, Hôpital Bicêtre, AP-HP Université Paris-Saclay, Inserm UMR S_999, Le Kremlin-Bicêtre, France; 21 Department of Critical Care, King’s College Hospital, London, UK; 22 Technical University of Munich, School of Medicine, Klinikum rechts der Isar, Department of Anesthesiology and Intensive Care Medicine, Munich, Bavaria Germany

**Keywords:** COVID-19, SARS-CoV-2, Early ambulation, Critical care, Intensive care units, Physical therapy specialty, Bed rest, Mobilisation

## Abstract

**Background:**

Early mobilisation (EM) is an intervention that may improve the outcome of critically ill patients. There is limited data on EM in COVID-19 patients and its use during the first pandemic wave.

**Methods:**

This is a pre-planned subanalysis of the ESICM UNITE-COVID, an international multicenter observational study involving critically ill COVID-19 patients in the ICU between February 15th and May 15th, 2020. We analysed variables associated with the initiation of EM (within 72 h of ICU admission) and explored the impact of EM on mortality, ICU and hospital length of stay, as well as discharge location. Statistical analyses were done using (generalised) linear mixed-effect models and ANOVAs.

**Results:**

Mobilisation data from 4190 patients from 280 ICUs in 45 countries were analysed. 1114 (26.6%) of these patients received mobilisation within 72 h after ICU admission; 3076 (73.4%) did not. In our analysis of factors associated with EM, mechanical ventilation at admission (OR 0.29; 95% CI 0.25, 0.35; *p* = 0.001), higher age (OR 0.99; 95% CI 0.98, 1.00; *p* ≤ 0.001), pre-existing asthma (OR 0.84; 95% CI 0.73, 0.98; *p* = 0.028), and pre-existing kidney disease (OR 0.84; 95% CI 0.71, 0.99; *p* = 0.036) were negatively associated with the initiation of EM. EM was associated with a higher chance of being discharged home (OR 1.31; 95% CI 1.08, 1.58; *p* = 0.007) but was not associated with length of stay in ICU (adj. difference 0.91 days; 95% CI − 0.47, 1.37,* p* = 0.34) and hospital (adj. difference 1.4 days; 95% CI − 0.62, 2.35,* p* = 0.24) or mortality (OR 0.88; 95% CI 0.7, 1.09, *p* = 0.24) when adjusted for covariates.

**Conclusions:**

Our findings demonstrate that a quarter of COVID-19 patients received EM. There was no association found between EM in COVID-19 patients' ICU and hospital length of stay or mortality. However, EM in COVID-19 patients was associated with increased odds of being discharged home rather than to a care facility.

*Trial registration* ClinicalTrials.gov: NCT04836065 (retrospectively registered April 8th 2021).

**Supplementary Information:**

The online version contains supplementary material available at 10.1186/s13613-023-01201-1.

## Introduction

Infection with the SARS-CoV-2 virus can lead to respiratory failure, requiring respiratory support and admission to an intensive care unit (ICU) [1]. Due to impaired muscle activity during critical illness, loss of muscle mass, muscle weakness, and functional limitations have been described in critically ill patients [2, 3]. The adverse impact of this so-called intensive care unit-acquired weakness (ICUAW) on weaning from mechanical ventilation and ICU and hospital length of stay are well-known [4, 5]. A Belgian single-centre study reported an incidence of ICUAW in coronavirus disease 2019 (COVID-19) patients of 52% at ICU discharge and 27% at hospital discharge [6]. COVID-19 patients affected by ICUAW had a median of 11-day longer ICU stay and low levels of mobilisation at ICU discharge, defined as being unable to sit independently at the edge of the bed. These significant functional limitations align with findings of another single-centre study from the United States investigating the outcome of COVID-19 patients after mechanical ventilation, which showed that 22% of patients required assistance with walking upon hospital discharge [7]. This occurred even though 94% of patients were functionally independent before admission to the hospital. Functional impairments of ICUAW can persist for years after discharge from ICU, leading to reduced quality of life, increased health care costs, and prolonged inability to work [4, 8]. Early mobilisation (EM) is an intervention that counteracts the described impairments. EM has demonstrated beneficial effects on functional independence and mobility at ICU discharge, resulting in shorter ICU and hospital lengths of stay and reduced duration of mechanical ventilation. Patients who received EM had a lower incidence of delirium and a higher likelihood of being discharged home [9, 10]. EM also revealed an improvement in the long-term outcome. It was observed that mobilised patients had less ICUAW and fewer long-term impairments one year after hospital discharge [11]. Due to this evidence, several guidelines recommended EM for all critically ill patients, provided there are no specific contraindications [12–14]. It is recognised that there is also the risk of adverse events with very intensive and active forms of EM [15]. However, as far as available, prevalence data demonstrate implementation rates between 0 and 33% [16–23].

With the SARS-CoV-2 pandemic starting in 2019, the number of patients with critical COVID-19 and severe acute respiratory syndrome increased rapidly [24]. At the pandemic's beginning, a patient population at high risk of developing ICUAW encountered healthcare systems trying to cope with limited bed capacity and staff resources [25]. Whether the COVID-19 pandemic impacted the implementation of EM is unknown.

The present study aimed to determine the implementation of EM in critically ill COVID-19 patients during the first wave of the pandemic in ICUs worldwide. Second, the study explored the factors associated with the implementation of EM and the outcomes of critically ill COVID-19 patients who underwent mobilisation.

## Methods

An international steering committee was established in 2020 by the European Society of Intensive Care Medicine (ESICM) to determine the burden of the novel COVID-19 disease in ICUs worldwide. ICUs were invited to participate in an international, multicenter, observational study (ESCIM UNITE-COVID study). The methodology and data collection have been extensively described by Greco et al. [25] in the first analysis and Conway Morris et al. [26]. The study was approved by the Ethics Committee of Ghent University Hospital (registration number BC07826) and received institutional approval at each participating site. The trial was registered at ClinicalTrials.gov (NCT04836065). The requirements for informed consent were compliant with local regulations. This study is a pre-planned subanalysis with a focus on EM. Data are available from 280 ICUs in 45 countries worldwide.

### Patients

Data were collected from patients who met all of the following inclusion criteria: (1) age 18 years or older; (2) admission to an ICU or another area in the hospital under the care of the intensive care team on the day of the ICU’s highest number of COVID-19 patients between February 15th and May 15th 2020; and (3) confirmed SARS-CoV2 infection by polymerase chain reaction or equivalent. Patients with SARS-CoV-2 infection but without COVID-19 diagnosis were excluded. In keeping with the observational study design, no additional interventions or measurements were performed, and patient care was delivered to local standards.

### Data management and extraction

Patient data were extracted from medical records from the day of admission up to day 60 of the ICU stay. Patient data were collected in the individual centres and submitted to a secure data-sharing platform (Clinfile, Vélizy-Villacoublay, France). The data were then curated according to the DAQCORD checklist; details were published previously [25]. The curation pipeline and code are publicly available on GitHub [27]. For our subanalysis, we excluded patients with missing data on EM (yes/no) and patients transferred from another ICU to avoid bias regarding the actual initiation of mobilisation.

### Variables

A priori, we selected relevant variables and cofactors for the analysis. To avoid any bias, we did not perform an imputation of missing data. Therefore, we excluded variables with more than 100 missing values to sustain an adequate number of observations. Control variables were then divided into: (1) demographics/admission data: sex, age, secondary comorbidities at admission (yes/no for each variable): chronic cardiac disease, chronic liver disease, history of hypertension, chronic neurological disease, chronic pulmonary disease, diabetes, asthma, malignant neoplasm, chronic kidney disease, immunosuppression, thromboembolic complications at admission, infection at admission, and country; (2) medications and supportive care during ICU stay at any point (yes/no for each variable) including antivirals, corticosteroids, antimalarial drugs, sedation, renal replacement therapy (RRT), inotropes/vasopressors, and tracheostomy.

### Outcomes

The primary outcome was the implementation of EM (yes/no) and influencing factors. EM included passive mobilisation, assisted-active mobilisation, and active mobilisation performed within 72 h after admission to the ICU, regardless of the duration [12, 28]. Passive mobilisation entailed at least the passive motions of all extremities in all physiological directions, passive cycling (bed pedal exerciser), passive vertical mobilisation (tilting table, standing frame), or passive transfer to rehabilitation chair. Positioning was not considered as early mobilisation. The level of mobility was assessed using the ICU Mobility Scale (IMS), a graded scale designed to document the highest level of mobility achieved by adult patients in an ICU [29]. The secondary outcomes that might be influenced by EM were the patient’s status 60 days after admission to the ICU (yes/no for each variable): death at any time point, discharged alive, still hospitalised, palliative discharge, transfer to another care facility and still in ICU. Furthermore, we looked at ICU and hospital length of stay (number of days). There are several clinical practice guidelines available for EM. We aimed to determine whether these guidelines impacted the implementation of EM. For each country, the percentage of patients receiving EM was determined and we explored if a national clinical practice guideline for EM was available.

### Statistical analysis

Statistical analyses were done in R [version R 4.1.1. (2021-08-10)]. Descriptive statistics: categorical variables are expressed as frequencies (percentages), and continuous variables are described with median and interquartile ranges. Significance testing for group differences was done with Chi-square tests for categorical data and Wilcoxon signed-rank test for continuous data using the tableone-package [30] and base R. For the primary and secondary analyses, we used mixed-effect multivariate linear and logistic models in combination with type III Anova using the car-package [31] and lme4-package [32]. Country was added as a random effect in all mixed models. To analyse factors associated with the initiation of EM (primary analyses), we built one multivariate mixed logistic model with EM (yes/no) as an outcome. For the secondary outcomes ICU and hospital length of stay, we used multivariate linear mixed models. For the secondary outcomes after 60 days, we built multivariate logistic mixed models for each endpoint separately. As described above, demographics, comorbidities, admission data, and medication and supportive care were included as covariates in the analyses of the secondary outcomes.

For primary analysis with EM as outcome, we only considered the effect of demographics, comorbidities and admission data; medication and supportive care received during the stay could have been received before or after the initiation of mobilisation since we did not record the exact dates and time of these interventions in our dataset. Therefore, we did not include them in the primary analysis. However, to present a comprehensive picture, we performed an association analysis comparing treatment differences and EM status.

The analysis considered guidelines identified through a systematic review by Lang et al. [33] and a literature search in PubMed using Medical Subject Headings (MeSH terms). Relevance was assessed based on title, abstract, and full text. Certain international mobilisation guidelines lacked specific country scopes, so country assignments were based on authors’ affiliations. Guidelines with defined scopes also aligned with this assignment approach. We used a t-test to analyse the effect of existing guidelines on EM rates and calculated a permutated *p-*value to account for heterogeneity and differences in sample sizes between countries.

A nominal alpha level of 0.05 was considered statistically significant.

## Results

**Fig. 1:**
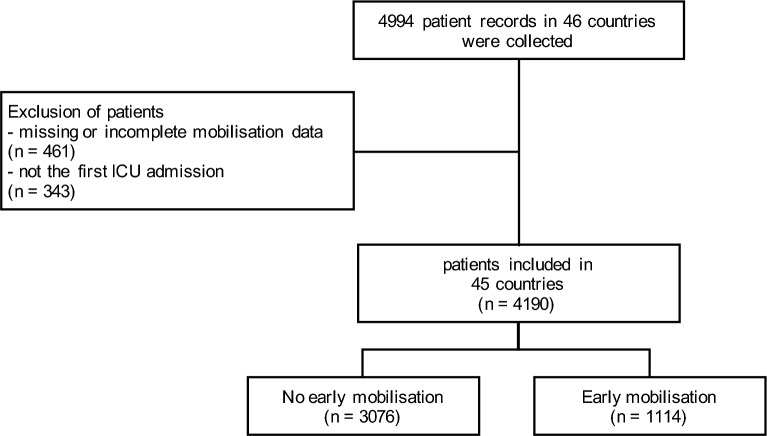
Flow diagram of available data, exclusion reasons, and missing data

**Fig. 2:**
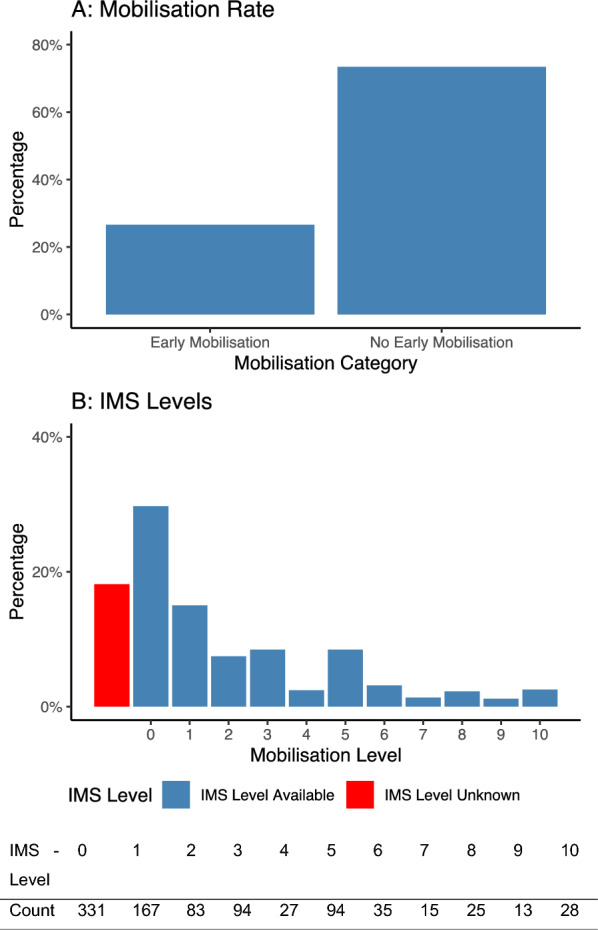
ICU Mobility Scale (IMS)—level and count in the EM group. **A** Illustrates the percentage distribution of the two groups receiving EM and no EM. **B** Shows the frequency of mobilisation levels achieved within the first 72 h after admission to the intensive care unit according to the ICU Mobility Scale. The x-axis displays the ICU Mobility Scale as an ordinal scale, and the y-axis illustrates the percentage of observations. The exact values are presented in the table below. IMS-Level: 0—Nothing (lying in bed), 1—Sitting in bed, exercises in bed, 2—Passively moved to chair (no standing), 3—Sitting over edge of bed, 4—Standing, 5—Transferring bed to chair, 6—Marching on spot (at bedside), 7—Walking with assistance of 2 or more people, 8—Walking with assistance of 1 person, 9—Walking independently with a gait aid, 10—Walking independently without a gait aid

In total, data from 4190 critically ill patients with COVID-19 admitted to 280 ICUs in 45 countries were analysed after excluding patients with missing mobilisation data (EM yes/no) or with secondary ICU admission to capture EM accurately (see Fig. 1). A comparison between the included patients and those excluded due to missing EM data can be found in Additional file 1: Table S1. 1114 patients (26.6%) received EM, with a median ICU Mobility Scale (IMS) of 1 [0, 4] (Fig. 1). 70.8% of patients were male, and the median age was 62 [54, 70]. The most common reason for ICU admission was respiratory failure due to critical COVID-19 (95.s8%), followed by other complications of COVID-19 (2.1%) or other diagnoses with coincident COVID-19 (2.0%). The most frequent comorbidity was arterial hypertension (50%). Baseline characteristics are shown in Table 1, and an overview of the number of included observations and missingness for each variable is given in Additional file 1: Table S2. The illustration shows the percentage distribution of the two groups receiving EM and no EM, along with the frequency of achieved levels of mobilization(Fig. 2).

**Table 1:** Patient demographics, admission data, and comorbidities at admission

	No EM *n* = 3076	EM *n* = 1114	*p*-value
Sex (male)	2176 (70.8)	798 (71.6)	0.61
Age	63 [54, 71]	60 [52, 69]	< 0.001
Body mass index	27.9 [25.3; 32.2]	28.0 [24.9; 32.2]	0.44
Chronic cardiac disease	464 (15.3)	179 (16.2)	0.49
Chronic liver disease	83 (2.7)	23 (2.1)	0.27
History of hypertension	1529 (50.3)	560 (50.4)	0.98
Chronic neurological disease	198 (6.5)	58 (5.2)	0.14
Chronic pulmonary disease	281 (9.3)	94 (8.5)	0.47
Diabetes	955 (31.5)	359 (32.4)	0.62
Asthma	269 (8.8)	80 (7.2)	0.10
Malignancy	165 (5.5)	60 (5.4)	0.99
Chronic kidney disease	232 (7.6)	66 (5.9)	0.07
Immunosuppression	154 (5.1)	51 (4.6)	0.55
Thromboembolic complications at admission	106 (3, 4)	48 (4, 3)	0.22
Additional infection at admission	440 (14.3)	163 (14.6)	0.84
Mechanical ventilation			< 0.001
Intubated at admission	1669 (54.9)	328 (30.3)	
Intubated during stay	1084 (35.7)	432 (39.9)	
Non-invasively ventilated during ICU stay	286 (9.4)	322 (29.8)	

*Data are presented as n (%) or median [IQR]*

### Early mobilisation

Variables present at admission that were associated with lower odds of initiating EM (see Additional file 1: Fig. S3) were mechanical ventilation at admission (OR 0.29; 95% CI 0.25, 0.35; *p* = 0.001), age (OR 0.99; 95% CI 0.98, 1.00; *p* =  ≤ 0.001), pre-existing asthma (OR 0.84; 95% CI 0.73, 0.98; *p* = 0.028) and pre-existing kidney disease (OR 0.84; 95% CI 0.71, 0.99; *p* = 0.036). The variable positively associated with the initiation of EM was cardiac disease (OR 1.14, 95% CI 1.01, 1.29; *p* < 0.030). There were differences in the type of supportive care and medications received during ICU admission when comparing patients receiving EM to those not receiving EM (Additional file 1: Table S3). The number of observations and missingness for each variable are provided in Additional file 1: Table S4. Countries with published mobilisation guidelines (*n* = 16) for critical care had significantly higher EM rates than countries without (*n* = 29; mean ± SD EM rate: 33 ± 25% vs 17 ± 16%, *p* = 0.031, respectively). Countries' EM rates are visualised in Additional file 1: Fig. S2, while details and guidelines are presented in Additional file 1: Table S5.

### Length of stay and 60-day outcomes

Overall, there was no significant difference in length of stay in ICU (adj. difference 0.91 days; 95% CI − 0.47, 1.37,* p* = 0.34) or hospital (adj. difference 1.4 days; 95% CI -0.62, 2.35,* p* = 0.24) when adjusted for covariates (Table 2).

**Table 2:** Outcomes

	No EM *n* = 3076	EM *n* = 1114	P (unadj.)	OR /${\varvec{\beta}}$ adjusted^a^ (95% CI)	P (adj.)^a^
ICU length of stay	18.00 [10.00, 28.00]	16.00 [8.00, 28.00]	0.001	0.91 (− 0.47, 1.37)	0.34
Hospital length of stay	30.00 [19.00, 45.00]	26.00 [16.00, 42.00]	< 0.001	1.4 (− 0.62, 2.35)	0.24
Outcome at 60 days					
Still in ICU	81 (2.6)	27 (2.4)	0.79	OR 0.76 (0.44, 1.32)	0.33
Still in hospital (not ICU)	137 (4.5)	62 (5.6)	0.16	OR 1.15 (0.79, 1.66)	0.47
Discharged home	1408 (45.8)	666 (59.8)	<0 .001	OR 1.31 (1.08, 1.58)	0.007
Transfer to other care facility ^b^	239 (7.8)	63 (5.7)	0.023	OR 0.69 (0.48, 0.98)	0.036
Palliative discharge	13 (0.4)	2 (0.2)	0.38	OR 0.11 (0.01, 1.54)	0.10
Deceased	1132 (36.8)	274 (24.6)	<0 .001	OR 0.88 (0.7, 1.09)	0.24

Data are presented as n (%) or median [IQR]. OR odds ratio; CI confidence interval. Sixty-six patients in the No EM cohort and 20 in the EM cohort with unknown/undefined/other outcomes. An overview of the count of included observations and missingness for each variable is given in Additional file 1: Table S6

^a^ The analysis was adjusted for the following variables: demographics/admission data: sex, age; secondary comorbidities at admission: chronic cardiac disease, chronic liver disease, history of hypertension, chronic neurological disease, chronic pulmonary disease, diabetes, asthma, malignant neoplasm, chronic kidney disease, immunosuppression, thromboembolic complications at admission, infection at admission, and country; medications and supportive care during ICU stay at any point: antivirals, corticosteroids, antimalarial drugs, sedation, renal replacement therapy (RRT), inotropes/vasopressors, and tracheostomy

^b^ Other care facility refers to institutions where patients are transferred after hospital if they could not independently care for themselves at home due to their health condition. This may include rehabilitation facilities or residential care facilities

**Fig. 3:**
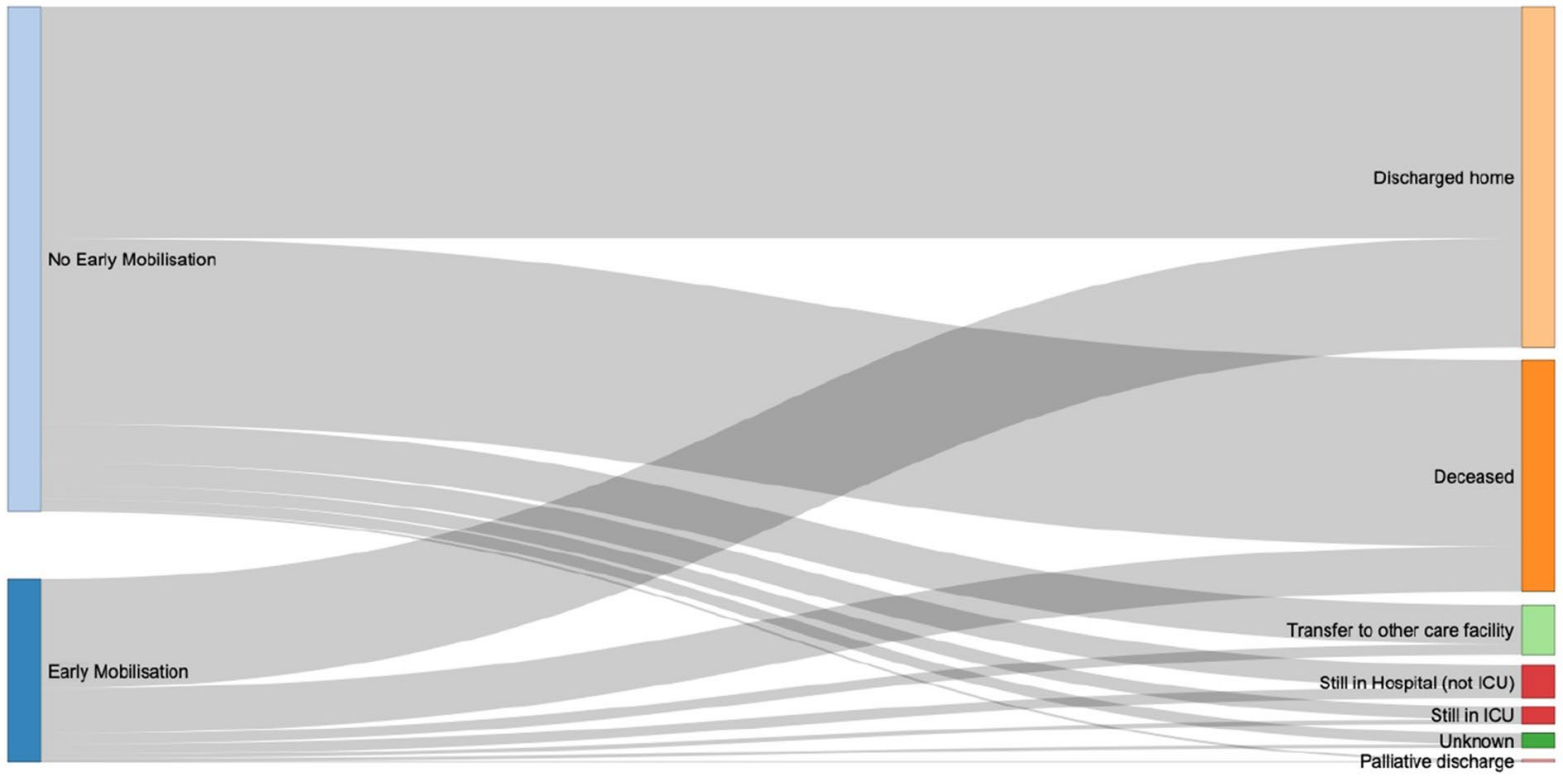
Sankey diagram. Sankey diagram representing the 60-day outcomes according to the No EM and EM group

There was no significant difference in mortality between patients receiving EM and those not receiving EM (OR 0.88; 95% CI 0.7, 1.09, *p* = 0.24) when adjusted for covariates. Patients who received EM were more likely to be discharged home than those not receiving EM (OR 1.31; 95% CI 1.08, 1.58, *p* = 0.007) and less likely to be discharged to another care facility (OR 0.69; 95% CI 0.48, 0.98,* p* = 0.036). In Fig. 3, the 60-day outcomes for the No EM and EM groups were visualised in a Sankey diagram.

## Discussion

This analysis of a large international cohort of COVID-19 patients in the ICU provides a reasonable estimate of the implementation of EM during the 1^st^ COVID-19 pandemic wave, with 27% of critically ill COVID-19 patients receiving mobilisation within the first 72 h of ICU admission. In addition, there were apparent differences in implementation; countries with published mobilisation guidelines for critical care had significantly higher EM rates. Initiation of EM was related to mechanical ventilation, age, and known comorbidities. Patients who received EM were more likely to be discharged home. Correspondingly, patients without EM were significantly more likely to be transferred to a care facility. However, we could not demonstrate a benefit of EM on length of stay or mortality.

Demographics and baseline comorbidities of our patient cohort were comparable to previous COVID-19 reports; the median age was 62 years, and male participants predominated. This is consistent with previous studies showing a higher risk of COVID-19 infection, more severe illness, and higher risk of ICU admission in men and older patients [34–36]. The most prevalent comorbidities were arterial hypertension and diabetes mellitus, similar to other studies [23, 37]. Respiratory failure was the main reason for admission to ICU (96%), resulting in a high intubation rate of 48% at ICU admission, again consistent with previous studies [34].

Günster et al. [36] analysed more than 8679 COVID-19 patients and showed a 26.8% readmission rate for any cause within 180 days of discharge and an increase in all-cause in-hospital mortality from 25 to 30% after 6 months. Consequently, the long-term effects of COVID-19 disease are serious. ICUAW was not recorded in these patients. Günster et al. showed that patients on mechanical ventilation, one of the main risk factors for ICUAW, had a higher readmission rate and worse outcomes. In the study by McWilliams et al. [23], all COVID-19 patients suffered from ICUAW at awakening. Frail patients had worse functional outcomes, which also influenced hospital discharge destination. The median time to first active mobilisation was 14 ± 7 days, i.e. there was no implementation of EM [23]. Pun et al. also reported on a cohort of COVID-19 patients from early 2020, noting a 33.9% incidence of mobilisation activities. However, this percentage refers only to days when patients were awake. When considering all patient days, the rate reduced to 16.7%, which is considerably lower than 26.6% in our cohort. Their study did not provide data on the initiation of mobilisation [38]. Studies in non-COVID-19 patients have demonstrated positive effects on outcomes if the intervention was started early [9, 10, 39]. This was further supported by a network-metaanalysis of non-COVID studies [28]. In COVID-19 patients, Schujmann et al. showed that timing of first mobilisation out-of-bed was an independent factor related to physical dependence after the ICU stay [40].

Our study provides international data with EM rates in different countries ranging from 0 to 100%. This information, however, must be interpreted with caution, given the different sample sizes per country. It is important to note that there are differences in healthcare organisation, post-hospital care, and therapeutic regimens across different countries, which can impact the measured outcomes. To account for this and to mitigate the impact, we included country information as a random effect in our models. For instance, the available personnel resources and the patient–staff ratios differed during the pandemic. Some studies suggested that a higher nurse-to-patient staffing ratio and the presence of trained physiotherapists were associated with more frequent and higher intensity mobilisation activities [19, 41]. The literature suggests that the availability of mobilisation protocols or published guidelines is a favourable predictor for implementing mobilisation in practice [22, 42–44]. Our study demonstrated a positive effect of existing guidelines specific to each country. The exact reasons behind the increased implementation rate resulting from the presence of national guidelines remain speculative. However, it is frequently observed that national guidelines are effectively launched and disseminated within the medical community of the respective country. One reason for better implementation of national guidelines might be related to the dissemination in the respective local language. Moreover, in some countries, national guidelines are considered quality indicators and failure to comply may have consequences for hospitals. We acknowledge that our EM rates might be biased to lower rates since this study used data from the peak day in each ICU, i.e. the day with the highest number of COVID-19 patients during the 1^st^ pandemic wave. This was most likely one of the days where workload was high for the critical care team members.

In our multivariate analysis, mechanical ventilation at admission was a strong predictor for lack of initiation of EM within the first 72 h. These results align with the findings of Liu et al. who reported high immobility rates of over 90% among mechanically ventilated patients in another large cohort during the pandemic, regardless of whether the patients had COVID-19 or not. In this study and also a recent study by Schellenberg et al. COVID-19 was not an independent factor hindering early mobilisation, but mechanical ventilation was [22, 45, 46]. In multiple studies conducted on nonCOVID-19 patients, mechanical ventilation has been identified as a barrier to early mobilisation before [16, 19–21, 44]. To overcome the barrier, a growing number of studies demonstrated the feasibility of EM in different settings, like protocols adapted to FiO2, positive end-expiratory pressure (PEEP), or other ventilator settings for mechanically ventilated patients [42, 47, 48].

There was no difference in mortality of length of stay between patients who received EM and those who did not receive EM. The mortality results are consistent with previous research, although some studies indicated a negligible effect on ICU and hospital length of stay in non-COVID-19 patients [10, 49–51]. Our study showed that patients who received EM were significantly more likely to be discharged home and less likely to be transferred to other care facilities. In general, the odds of being transferred to a care facility increased with limited mobility or muscle strength [52]. This is consistent with the evidence from non-COVID-19 data showing a link between functional or mobility status and a higher rate of being discharged home [50, 51, 53, 54].

To the best of our knowledge, this is the most extensive study with data from 45 countries. Since there are no uniform criteria for EM, we used a definition based on one guideline and high-level evidence [12, 28, 55]. The study was conducted during the first wave of the pandemic when many uncertainties existed. We chose the day when each ICU had the peak number of patients. It follows that the study provides insight into mobilisation practices at the time of maximum ICU burden. Understandably, there are limitations in data collection during this particular period. Specific details were not captured, including frailty scores, delirium, and traditional severity scores such as SOFA and APACHE. The analysis was adjusted for supportive therapy provided, which depended on the severity of organ failure. However, functional outcomes following ICU and hospital stay are unavailable due to the absence of long-term patient follow-up. Regarding our statistical methods, the lack of specific timings for initiation of medications and supportive care in our dataset precluded their inclusion in the primary early mobilisation analysis, presenting a study limitation.

This contrasts with the apparent strength of the study; gaining insight under such unfavourable conditions was a scarce opportunity; a large number of patients were included at study sites worldwide. Until now, it is one of the most extensive data sets on COVID-19 patients in ICUs and the implementation of EM in general.

## Conclusion

Our findings demonstrate that a quarter of critically ill COVID-19 patients received EM worldwide during the 1st pandemic wave of a novel viral pandemic. EM was not associated with ICU and hospital length of stay, nor with mortality when adjusted for the covariates. However, EM was associated with increased odds of being discharged home rather than to a care facility.

### Supplementary Information

**Additional file 1: Figure S1.** Multivariate model of factors influencing EM. **Figure S2.** Percentage of patients receiving EM in countries with at least 10 included patients. **Table S1.** Comparison of included patients with patients that were excluded based on missing EM data. **Table S2.** Variables with the count of missing data and number of observations in the analysis of patient demographics, admission data and comorbidities at admission. **Table S3.** Supportive care and medications during the stay in ICU. **Table S4.** Variables with the count of missing data and included observations in the analysis supportive care and medications during the stay. **Table S5.** Countries with percentage of patients receiving EM, patient count and clinical practice guidelines (based on the systematic review of lang et al. (1) and pub-med search). **Table S6.** Variables with the count of missing data and included observations in the analysis of the 60 day outcomes.

## Data Availability

The datasets used and/or analysed during the current study are available from the corresponding author on reasonable request. The data curation pipeline and data quality assessment (version 3.1) are publicly available https://doi.org/10.5281/zenodo.6063905.

## Abbreviations

COVID-19: Coronavirus disease 2019
ECMO: Extracorporeal membrane oxygenation
EM: Early mobilisation
HFNC: High-flow nasal cannula
ICU: Intensive care unit
ICUAW: Intensive care unit acquired weakness
IMS: ICU Mobility scale
NIV: Non-invasive ventilation
PEEP: Positive and end-expiratory pressure
RRT: Renal replacement therapy
SARS-CoV-2: Severe acute respiratory syndrome coronavirus type 2
