# Supplementary material for: Early mobilisation in critically ill COVID-19 patients: a subanalysis of the ESICM-initiated UNITE-COVID observational study
Source: Ann Intensive Care. 2023 Nov 14;13:112. doi: 10.1186/s13613-023-01201-1 (PMC10645963; doi:10.1186/s13613-023-01201-1)
Supplement: Supplementary file 1 — Additional file 1: Figure S1. Multivariate model of factors influencing EM. Figure S2. Percentage of patients receiving EM in countries with at least 10 included patients. Table S1. Comparison of included patients with patients that were excluded based on missing EM data. Table S2. Variables with the count of missing data and number of observations in the analysis of patient demographics, admission data and comorbidities at admission. Table S3. Supportive care and medications during the stay in ICU. Table S4. Variables with the count of missing data and included observations in the analysis supportive care and medications during the stay. Table S5. Countries with percentage of patients receiving EM, patient count and clinical practice guidelines (based on the systematic review of lang et al. (1) and pub-med search). Table S6. Variables with the count of missing data and included observations in the analysis of the 60 day outcomes. [file 13613_2023_1201_MOESM1_ESM.pdf]

Electronic supplementary material (ESM) – Additional File 1

Early mobilisation in critically ill COVID-19 patients: A subanalysis of the ESICM-initiated UNITE-COVID observational study.

Philipp Kloss, Maximilian Lindholz, Annette Milnik, Elie Azoulay, Maurizio Cecconi, Giuseppe Citerio, Thomas De Corte, Frantisek Duska, Laura Galarza, Massimiliano Greco, Armand R J Girbes, Jozef Kesecioglu, Johannes Mellinghoff, Marlies Ostermann, Mariangela Pellegrini, Jean-Louis Teboul, Jan De Waele, Adrian Wong, Stefan J Schaller

Content

SUPPLEMENTARY FIGURES 2

Supplementary Figure S1. Multivariate model of factors influencing EM 2

Supplementary Figure S2. Percentage of patients receiving EM in countries with at least 10 included patients. 3

SUPPLEMENTARY TABLES 4

Supplementary Table S1. Comparison of included patients with patients that were exluded based on missing EM data 4

Supplementary Table S2. Variables with the count of missing data and number of observations in the analysis of patient demographics, admission data and comorbidities at admission 5

Supplementary Table S3. Supportive care and medications during the stay in ICU 6

Supplementary Table S4. Variables with the count of missing data and included observations in the analysis supportive care and medications during the stay 7

Supplementary Table S5. Countries with percentage of patients receiving EM, patient count and clinical practice guidelines (based on the Systematic Review of Lang et al. (1) and pub-med search). 8

Supplementary Table S6. Variables with the count of missing data and included observations in the analysis of the 60 day outcomes 9

REFERENCES 10

Supplementary Figures

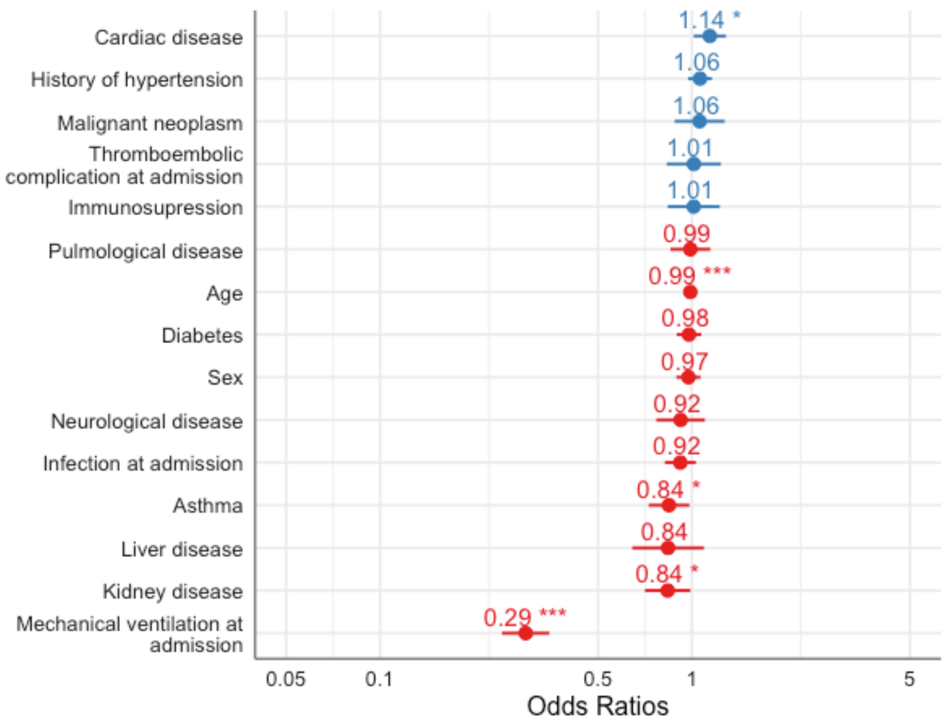

Supplementary Figure S1. Multivariate model of factors influencing EM

The Forest plot shows the results of multivariate regression analysis for factors influencing early mobilisation. The x-axis represents the Odds ratio on a log scale with the Odds ratios (dots) and 95% CI (whiskers).

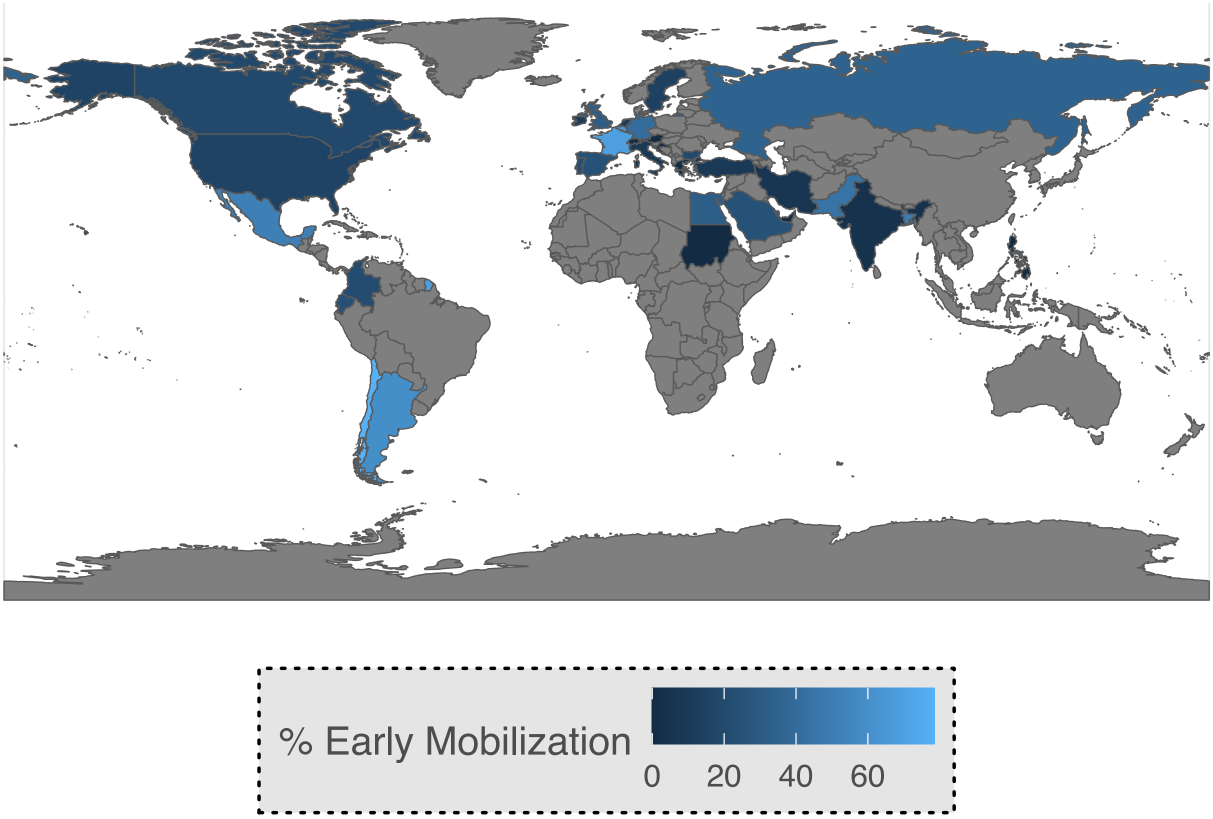

Supplementary Figure S2. Percentage of patients receiving EM in countries with at least 10 included patients.

The number of mobilized patients represented using a color scale (for exact numbers see Supplementary Table S4). The countries shaded in grey did not participate in the study.

Supplementary Tables

Supplementary Table S1. Comparison of included patients with patients that were exluded based on missing EM data
(not patients with referral from other ICU, the first exclusion reason)

	No missing EM data n = 4190	Missing EM data n = 263	*p*-value
Sex (Male)	2974 (71.0)	188 (72.0)	0.77
Age	62 [54, 70]	61 [53, 70]	0.80
Body Mass Index	29.4 (6.22)	29.1 (7.04)	0.63
Chronic cardiac disease	643 (15.5)	46 (18.4)	0.25
Chronic liver disease	106 (2.6)	10 (4.0)	0.22
History of hypertension	2089 (50.3)	114 (45.6)	0.16
Chronic neurological disease	256 (6.2)	11 (4.5)	0.34
Chronic pulmonary disease	375 (9.0)	30 (12.0)	0.15
Diabetes	1314 (31.7)	92 (36.4)	0.14
Asthma	349 (8.4)	29 (11.6)	0.10
Malignancy	225 (5.5)	16 (6.6)	0.54
Chronic kidney disease	298 (7.2)	17 (6.8)	0.93
Immunosuppression	205 (5.0)	13 (5.3)	0.92
Thromboembolic complications at admission	4036 (96.3)	254 (96.6)	0.96
Additional infection at admission	603 (14.4)	38 (15.8)	0.60
Mechanical ventilation	2947 (72.7)	147 (69.0)	0.27

Supplementary Table S2. Variables with the count of missing data and number of observations in the analysis of patient demographics, admission data and comorbidities at admission

	No EM n = 3076		EM n = 1114	
	Count of missing data	Count of observations	Count of missing data	Count of observations
Sex	1	3075	0	1114
Age	8	3068	2	1112
Body Mass Index	226	2850	94	1020
Chronic cardiac disease	37	3039	9	1105
Chronic liver disease	48	3028	4	1110
History of hypertension	36	3040	3	1111
Chronic neurological disease	44	3032	6	1108
Chronic pulmonary disease	41	3035	5	1109
Diabetes	45	3031	5	1109
Asthma	32	3044	4	1110
Malignant neoplasm	74	3002	7	1107
Chronic kidney disease	33	3043	3	1111
Immunosuppression	72	3004	8	1106
Thromboembolic complications at admission	0	3076	0	1114
Additional infection at admission	5	3071	0	1114
Mechanical ventilation	37	3039	32	1082

Supplementary Table S3. Supportive care and medications during the stay in ICU (unadjusted differences)

	No EM<br>n = 3076	EM<br>n = 1114	*p*-value
Treatment drugs			
Antivirals	1434 (46.9)	450 (40.7)	<.001
Corticosteroids	1609 (52.7)	539 (48.6)	.021
Antimalaria medication	1899 (61.8)	610 (54.8)	<.001
Therapeutic anticoagulation	1290 (42.1)	477 (42.9)	.65
Antibiotics	2719 (88.5)	963 (86.4)	.07
Antifungals	187 (6.1)	55 (4.9)	.18
Supportive treatments			
Sedation	2746 (89.3)	800 (71.8)	<.001
RRT	762 (24.8)	219 (19.7)	.001
Inotropes/Vasopressors	2459 (79.9)	696 (62.5)	<.001
Neuromuscular blockers	2172 (71.3)	626 (56.5)	<.001
Ventilatory Support			
Tracheostomy	923 (30.1)	333 (30.0)	1.00
NIV	605 (19.8)	277 (25.0)	<.001
HFNC	467 (15.3)	309 (28.0)	<.001
ECMO	76 (2.5)	31 (2.8)	.66
Prone positioning	1901 (62.9)	669 (60.4)	.14

Data are presented as n (%). *RRT* Renal replacement therapy, *NIV* non-invasive ventilation, *HFNC* high-flow nasal cannula, *ECMO* extracorporeal membrane oxygenation

Supplementary Table S4. Variables with the count of missing data and included observations in the analysis supportive care and medications during the stay

	No EM n = 3076		EM n = 1114	
	Count of missing data	Count of observations	Count of missing data	Count of observations
Treatment drugs				
Antivirals	21	3055	7	1107
Corticosteroids	23	3053	5	1109
Antimalaria medication	3	3073	1	1113
Therapeutic anticoagulation	11	3065	3	1111
Antibiotics	5	3071	0	1114
Antifungals	3	3073	0	1114
Supportive treatments				
Sedation	1	3075	0	1114
RRT	4	3072	0	1114
Inotropes/Vasopressors	0	3076	0	1114
Neuromuscular blockers	28	3048	6	1108
Tracheostomy	6	3070	4	1110
NIV	23	3053	7	1107
HFNC	27	3049	12	1102
ECMO	12	3064	1	1113
Prone positioning	56	3020	6	1108

Supplementary Table S5. Countries with percentage of patients receiving EM, patient count and clinical practice guidelines (based on the Systematic Review of Lang et al. (1) and pub-med search).

Country	Percentage of patients receiving EM	Patient count	Authors of a Clinical Practice Guideline	Reference
Nigeria	100	9	No / not known	
Romania	80	5	No / not known	
Chile	78	51	Yes	(2)
France	68	67	Yes	(3)
Belgium	64	190	Yes	(4), (5)
Argentina	60	10	Yes	(2), (6)
Mexico	51	112	Yes	(2)
Bangladesh	50	16	No / not known	
Pakistan	44	36	No / not known	
Germany	41	95	Yes	(7), (5)
Russia	35	210	No / not known	
Egypt	32	112	No / not known	
United Kingdom	31	646	Yes	(5)
Estonia	28	7	No / not known	
Qatar	26	233	No / not known	
Singapore	26	23	No / not known	
Saudi Arabia	25	32	No / not known	
Spain	24	728	Yes	(8)
Ecuador	23	63	No / not known	
Portugal	22	152	No / not known	
Colombia	21	42	Yes	(2)
Bulgaria	20	10	No / not known	
Canada	19	36	Yes	(3), (5), (9), (8)
United States	15	184	Yes	(2), (3), (9), (8)
Sweden	14	89	No / not known	
Netherlands	11	315	Yes	(3), (4), (5)
Ireland	11	35	Yes	(5)
Turkey	9	249	No / not known	
Italy	8	234	Yes	(5)
Iran	6	29	No / not known	
India	4	22	No / not known	
Greece	0	30	No / not known	
Croatia	0	26	No / not known	
Switzerland	0	19	No / not known	
Philippines	0	15	No / not known	
United Arab Emirates	0	10	No / not known	
Sudan	0	10	No / not known	
Austria	0	10	Yes	(7)
Suriname	0	9	No / not known	
Morocco	0	6	No / not known	
Libya	0	5	No / not known	
Peru	0	2	No / not known	
Kenya	0	2	No / not known	
Brazil	0	2	Yes	(10)
Norway	0	1	No / not known	

Supplementary Table S6. Variables with the count of missing data and included observations in the analysis of the 60 day outcomes

	No EM n = 3076		EM n = 1114	
	Count of missing data	Count of observations	Count of missing data	Count of observations
ICU length of stay	181	2895	55	1059
Hospital length of stay	1469	1607	405	709
Outcome at 60 day				
Still in ICU	0	3076	0	1114
Still in Hospital (not ICU)	0	3076	0	1114
Discharged home	0	3076	0	1114
Transfer to other facility	0	3076	0	1114
Palliative discharge	0	3076	0	1114
Deceased	0	3076	0	1114

References

1. Lang JK, Paykel MS, Haines KJ, Hodgson CL. Clinical Practice Guidelines for Early Mobilization in the ICU: A Systematic Review. Crit Care Med. 2020;48(11):e1121-e8.

2. Celis-Rodríguez E, Díaz Cortés JC, Cárdenas Bolívar YR, Carrizosa González JA, Pinilla DI, Ferrer Záccaro LE, et al. Evidence-based clinical practice guidelines for the management of sedoanalgesia and delirium in critically ill adult patients. Med Intensiva (Engl Ed). 2020;44(3):171-84.

3. Devlin JW, Skrobik Y, Gélinas C, Needham DM, Slooter AJC, Pandharipande PP, et al. Clinical Practice Guidelines for the Prevention and Management of Pain, Agitation/Sedation, Delirium, Immobility, and Sleep Disruption in Adult Patients in the ICU. Crit Care Med. 2018;46(9):e825-e73.

4. Sommers J, Engelbert RH, Dettling-Ihnenfeldt D, Gosselink R, Spronk PE, Nollet F, et al. Physiotherapy in the intensive care unit: an evidence-based, expert driven, practical statement and rehabilitation recommendations. Clin Rehabil. 2015;29(11):1051-63.

5. Gosselink R, Bott J, Johnson M, Dean E, Nava S, Norrenberg M, et al. Physiotherapy for adult patients with critical illness: recommendations of the European Respiratory Society and European Society of Intensive Care Medicine Task Force on Physiotherapy for Critically Ill Patients. Intensive Care Med. 2008;34(7):1188-99.

6. Donato M, Carini FC, Meschini MJ, Saubidet IL, Goldberg A, Sarubio MG, et al. Consensus for the management of analgesia, sedation and delirium in adults with COVID-19-associated acute respiratory distress syndrome. Rev Bras Ter Intensiva. 2021;33(1):48-67.

7. Th. Bein MB, U. Brückner, K. Gebhardt, D. Henzler, C. Hermes, K., Lewandowski MM, M. Nothacker, Th. Staudinger, M. Tryba, S. Weber-Carstens, H., Wrigge. S2e-Leitlinie: „Lagerungstherapie und Frühmobilisation zur Prophylaxe oder Therapie von pulmonalen Funktionsstörungen". 2015.

8. Girard TD, Alhazzani W, Kress JP, Ouellette DR, Schmidt GA, Truwit JD, et al. An Official American Thoracic Society/American College of Chest Physicians Clinical Practice Guideline: Liberation from Mechanical Ventilation in Critically Ill Adults. Rehabilitation Protocols, Ventilator Liberation Protocols, and Cuff Leak Tests. Am J Respir Crit Care Med. 2017;195(1):120-33.

9. Murray MJ, DeBlock H, Erstad B, Gray A, Jacobi J, Jordan C, et al. Clinical Practice Guidelines for Sustained Neuromuscular Blockade in the Adult Critically Ill Patient. Crit Care Med. 2016;44(11):2079-103.

10. Aquim EE, Bernardo WM, Buzzini RF, Azeredo NSG, Cunha LSD, Damasceno MCP, et al. Brazilian Guidelines for Early Mobilization in Intensive Care Unit. Rev Bras Ter Intensiva. 2019;31(4):434-43.
